# Working Memory Alterations After a Romantic Relationship Breakup

**DOI:** 10.3389/fnbeh.2021.657264

**Published:** 2021-04-09

**Authors:** Anne M. Verhallen, Remco J. Renken, Jan-Bernard C. Marsman, Gert J. ter Horst

**Affiliations:** Cognitive Neuroscience Center, Department of Biomedical Sciences of Cells and Systems, University Medical Center Groningen, Groningen, Netherlands

**Keywords:** working memory, *n*-back task, task-based fMRI, depressive symptoms, relationship breakup

## Abstract

Experiencing stress can have a disadvantageous effect on mental well-being. Additional to the relation between suffering from chronic stress and depression, both stress (acute and chronic) and depression are associated with cognitive alterations, including working memory. The breakup of a relationship is considered to be a stressful event that can lead to symptoms of depression in otherwise healthy people. Additional to elevated depression scores, stress-related cognitive alterations may occur in this population as well. Therefore, in the present fMRI study we investigated whether experiencing a relationship breakup is associated with working memory alterations and whether this is related to depressive symptom severity. A three workload version of the *n*-back task (0-back, 1-back, 2-back) was used to measure working memory in subjects who experienced a breakup in the preceding 6 months (“heartbreak group”, *n* = 70) and subjects in a romantic relationship (“relationship group”, *n* = 46). Behavioral task performance was compared between the two groups. Functional MRI scans were analyzed using General Linear Model (GLM) activation analyses. Workload conditions were contrasted to each other and to baseline and group differences were assessed. To investigate whether brain networks are associated with depressive symptom severity within the heartbreak group specifically, a *post hoc* feature-based Independent Component Analysis was performed on the 2-back > 0-back contrast images to identify brain regions that covaried across subjects. Behaviorally, the heartbreak group performed similar at high workload (i.e., 2-back) and better at moderate workload (i.e., 1-back) than the relationship group. GLM analysis revealed an interaction between group and 2-back > 0-back, 2-back > 1-back and 2-back > baseline; the heartbreak group showed less precuneus activation compared to the relationship group. Furthermore, within the heartbreak group, we found a negative association between depressive symptom severity and a brain network representing mostly the precuneus, anterior cingulate gyrus and supplementary motor cortex. Our findings suggest that the effect of a breakup is accompanied by workload-dependent working memory alterations. Therefore, we propose that this population can potentially be used to investigate the interplay between stress, cognitive functioning and depression.

## Introduction

Experiencing stress can have a disadvantageous effect on mental well-being. Stressful life-events are considered risk factors for developing symptoms of depression (Kendler et al., 1999). Furthermore, chronic stress and dysregulation of the stress response can lead to mood disorders, including depression (Bale, 2006). Additional to the relation between suffering from chronic stress and depression, both stress and depression are associated with cognitive alterations.

Acute stress as well as chronic stress is known to have an impairing effect on working memory functioning in healthy individuals. This effect can be present at either the behavioral level, neural level or both. For example, acute stress, induced by watching a movie with aversive violent content, led to decreased dorsolateral prefrontal cortex activation during performance of a working memory task in a healthy female sample, but not to aberrant behavioral performance (Qin et al., 2009). Another study, investigating the effect of executing a social stress test in male subjects, revealed that acute social stress reduced performance (slower reaction times and lower accuracy rates) at high workload (Schoofs et al., 2008). Furthermore, animal studies showed that chronic stress, induced by a model of restraint stress, resulted in a variety of neural, and associated cognitive capacity, alterations, including working memory impairment (Grizzell et al., 2014). These stress-induced cognitive impairments were found to be accompanied by structural changes in the prefrontal cortex (Cerqueira et al., 2007). In humans, suffering from chronic psychological stress was found to be associated with lowered cognitive abilities, including working memory (Mackenzie et al., 2009). Additionally, impaired cognitive performance on executive functioning related tasks was found in people suffering from severe work-related chronic stress (Ohman et al., 2007) and people who are on sick leave due to work stress showed lowered activation in prefrontal brain areas (at similar behavioral performance) during a working memory task (Sandström et al., 2012).

Cognitive disturbances, especially at the executive domain, are common in depression as well. Patients diagnosed with major depression disorder displayed worse executive functioning, including attention and working memory, compared to healthy controls (Cotrena et al., 2016). In addition, depressed subjects showed reduced performance on a working memory task in comparison with a healthy control group (Rose and Ebmeier, 2006). The study by Harvey et al. (2004) elucidates especially impairments regarding updating of information among patients diagnosed with major depression disorder. At the neural level, both hyperactivation and hypoactivation of task-related brain areas was found in various neuroimaging studies. For example, greater activation in right prefrontal brain areas during performance of two working memory tasks (Tower of London and *n*-back) was found in depressed patients in comparison with a healthy control group in an fMRI study (Fitzgerald et al., 2008). At the behavioral level, results were less clear: differences between the two groups were found only for the Tower of London task (Fitzgerald et al., 2008). Similar findings were present in a study by Harvey et al. (2005) and a study by Matsuo et al. (2007): depressed patients performed comparable to healthy controls at the behavioral level, while greater activation of the prefrontal cortex and anterior cingulate cortex was found. In a study by Rose et al. (2006), hyperactivation was found in the medial orbitofrontal cortex/rostral anterior cingulate cortex at similar performance in terms of accuracy and reaction time. Contradictory, hypoactivation in several brain regions, such as the left dorsolateral prefrontal cortex and the precuneus, during working memory performance was found in depressed patients compared to healthy controls (Rodríguez-Cano et al., 2014). In a study by Korgaonkar et al. (2013), hypoactivation in the right dorsolateral prefrontal cortex was found, accompanied by hyperactivation in the anterior cingulate cortex. Taken together, even though stress and depression seem to be associated with working memory alterations, previous studies have reported diverse results with regard to the affected level (i.e., either behavioral, neural or both).

In our laboratory, we explore whether the dissolution of a romantic relationship can be used as an experimental human model to examine depressive symptomatology following a stressful event in a population without a psychiatric disorder. Results from a questionnaire study in our laboratory, administered to the same study population as presented in the present paper, display elevated depression scores, albeit subclinical, in individuals who have experienced a romantic relationship dissolution compared to a reference group consisting of individuals in a romantic relationship (Verhallen et al., 2019). In addition, high depression scores after breakup were associated with altered resting-state functional connectivity patterns in our laboratory (Alonso Martínez et al., 2020). The finding of elevated depression scores following relationship breakup is complemented by previous results from other laboratories displaying an association between suffering from a romantic relationship breakup and symptoms of depression (Monroe et al., 1999; Field et al., 2009; Stoessel et al., 2011). For example, Stoessel et al. (2011) found that all of the included participants, with a relationship breakup in the preceding 6 months and still feeling sad about the breakup, scored within the range of clinical depression on a rating scale measuring severity of depressive symptoms. Monroe et al. (1999) even showed an association between the occurrence of a relationship breakup and first onset of major depression in a young population. Based on these psychological findings we already proposed that the breakup of a romantic relationship could be used as an experimental model to study a depression-like state in people without a psychiatric disorder (Verhallen et al., 2019).

It might be that, additional to elevated depression scores, stress-related cognitive alterations occur in this population as well. Therefore, in the present study we investigated whether experiencing a romantic relationship breakup is associated with working memory alterations, both at the neural and behavioral level. Additionally, we investigated whether the possible association between working memory functioning and relationship breakup is related to depressive symptom severity. To this end, we invited subjects who experienced a romantic relationship breakup within the preceding 6 months (the “heartbreak group”) and subjects in a romantic relationship (the “relationship group”) to participate in our fMRI study, including performing a three workload version of the *n*-back task. The *n*-back task is a frequently used paradigm in fMRI studies to assess working memory performance at alternating workload conditions (Kirchner, 1958). Storing information as well as updating information is required in order to perform this task adequately. From previous research, it is known that especially frontal and parietal brain areas are involved in performing the *n*-back task. A meta-analysis by Owen et al. (2005) revealed six brain regions consistently involved in healthy individuals: dorsal cingulate and medial premotor cortex, bilateral premotor cortex, dorsolateral and ventrolateral prefrontal cortex, medial and lateral posterior parietal cortex and the frontal poles. In addition, Emch et al. (2019) showed that cortical areas (frontal and parietal) as well as subcortical areas, such as the insula, and the cerebellum are activated during verbal working memory task performance. The research aim of the present study had an explorative nature, as previous studies have reported diverse results. We expected working memory alterations in people who experienced a romantic relationship breakup compared to people in a romantic relationship. Since both hyperactivation and hypoactivation at the neural level (Harvey et al., 2005; Rose et al., 2006; Matsuo et al., 2007; Fitzgerald et al., 2008; Sandström et al., 2012; Korgaonkar et al., 2013; Rodríguez-Cano et al., 2014), sometimes accompanied by inferior behavioral performance (Harvey et al., 2004; Ohman et al., 2007; Schoofs et al., 2008; Mackenzie et al., 2009), was found in stressed and/or depressed populations, we were not able to hypothesize on the direction of these breakup-related effects.

## Materials and Methods

### Study Design

71 subjects and 46 subjects were included in, respectively, the heartbreak group and the relationship group between 2011 and 2013. The complete study consisted of an fMRI paradigm and a questionnaire battery. The following tasks were included in our fMRI paradigm in this order: picture task, first run *n*-back task, anatomical scan, rumination task, second run *n*-back task and resting state. In the picture task, pictures of the (ex-) partner and an age-and gender-matched acquaintance were presented, similar to the study by Fisher et al. (2010). In the rumination task, subjects were instructed to ruminate about their (ex-) partner, similar to the study by Najib et al. (2004). Two *n*-back task runs were included in our paradigm in order to explore potential rumination-related effects. In this paper, results for the first *n*-back task are presented only, as we merely aimed to examine working memory alterations. Questionnaire battery results are presented in the paper from Verhallen et al. (2019).

Written informed consent was obtained from every subject. The study was approved by the Medical Ethical Committee of the University Medical Center Groningen and conducted in accordance with the principles of the Declaration of Helsinki. Subjects received a financial compensation for their invested time.

### Study Population

Men and women who experienced a romantic relationship breakup within the preceding 6 months (relationship duration prior to breakup of at least 6 months) were included in the heartbreak group. Men and women in a romantic relationship were included in the relationship group. In the relationship group, relationship duration had to be at least 6 months, as stress hormone levels were found to be elevated during the first period of a new relationship (Marazziti and Canale, 2004), with a maximum of 24 months to include a homogeneous group in terms of relationship stage (Hatfield and Sprecher, 1986). Further details of our study population (recruitment strategy, inclusion-and exclusion criteria) are described in the paper from Verhallen et al. (2019). One subject of the heartbreak group could not be included in the present analysis, because of missing logfiles for the *n*-back task.

### Experimental Paradigm

Before the start of the fMRI paradigm, a questionnaire battery, including demographic and mood-related information, was administered. Depression scores were revealed for both groups using a Dutch version of the Major Depression Inventory (MDI). The MDI is a validated questionnaire to assess severity of depressive symptoms, based on the DSM-IV and ICD-10 diagnostic criteria (Bech et al., 2001, 2015). Demographics and MDI scores fall within the scope of the present paper.

A block design three workload version of the *n*-back task (0-back, 1-back, and 2-back) was used to assess verbal working memory functioning at both the behavioral and neural level. We used E-prime stimulus software (Psychology Software Tools, Pittsburgh, PA, United States) to present the task. Table 1 shows the sequence of the alternating workload conditions. Seven or eight letters were one at one randomly presented in each condition for 500 milliseconds. A black screen appeared between each stimulus for 1,500 milliseconds. Each condition was presented four times during a single run of the task. In total, 30 letters were presented for each condition. After finishing each block, a fixation cross (FC) appeared for 10 s. An instruction screen was presented for 3 s before each condition in every block. Subjects received written information about the task, including a link to a recommended website to practice in advance. At the day of the experiment, subjects could practice outside the scanner on a laptop to get familiar with the task. At the 0-back condition, subjects were instructed to press a button whenever a pre-specified letter appeared on the screen. At the 1-back condition and 2-back condition, subjects had to press a button whenever the presented letter matched the letter, respectively, one or two letters before. The duration time of the *n*-back task was approximately 4 min and 30 s. For each subject 134 volumes were included in the present analysis.

**TABLE 1:** Sequence alternating workload conditions across the task.

Block	1	2	3	4
**First *n*-back condition presented**	0	1	2	2
**Second *n*-back condition presented**	1	0	0	1
**Third *n*-back condition presented**	2	2	1	0
**Baseline**	FC	FC	FC	FC

### Image Acquisition

Images were obtained with a 3 Tesla Philips Intera MRI scanner with a 32-channel head coil (Philips Medical Systems, Best, Netherlands). A T1-weighted anatomical scan of the whole brain was acquired for every subject with the following scanning parameters: slice thickness 1 mm; voxel size 1 × 1 × 1 mm, 170 slices; field of view (AP × FH × RL) 232 × 170 × 256 mm; flip angle 8°). Functional Echo Planar Imaging (EPI) images were acquired with the following scanning parameters: repetition time (TR) 2,000 ms; echo time (TE) 30 ms; slice thickness 3.5 mm; voxel size 3.5 × 3.5 × 3.5 mm; 37 slices; field of view (AP × FH × RL) 224 × 129.5 × 224 mm; flip angle 70°).

### Processing of Behavioral Data

Behavioral data were processed and analyzed using IBM SPSS Statistics 24 and visualized using RStudio version 1.2.1335.

#### Outcome Measures

Accuracy rates and reaction times were obtained as an outcome measure of task performance at the behavioral level. “Accuracy” represents the average percentage of correct responses across the four blocks for each workload condition. “Reaction time” (RT) represents the average number of milliseconds to respond to a correct match across the four blocks for each workload condition. Task performance data (0-back accuracy, 1-back accuracy, 2-back accuracy, 0-back RT, 1-back RT, and 2-back RT) were derived from the E-prime logfiles. Depression scores were calculated by summing the scores of the individual MDI items according to the scoring guidelines for using MDI as a rating scale to assess severity of depressive symptoms and theoretically range between 0 and 50 (Bech et al., 2015).

#### Statistical Analysis

To check if our behavioral data were normally distributed, a Shapiro Wilk test for normality was used. Because the distribution of our data was found to be skewed, non-parametrical statistical tests were performed in further analyses. Group differences with regard to demographics, accuracy rates and reaction times were assessed with a Mann-Whitney *U* test. Spearman rank tests were performed to test whether MDI scores correlated with accuracy and RT. Behavioral results were considered significant at *p*-value < 0.05 (uncorrected).

### Processing of fMRI Data

fMRI data were processed and analyzed using SPM12 (Wellcome Trust Centre for Neuroimaging^1^) and the GIFT toolbox v4.0b, running in Matlab R2015a (The MathWorks Inc., Natick, MA, United States). In addition, we used FSLeyes version 0.10.1b for visualization purposes.

#### Preparation and Preprocessing

The raw MR images were first converted to NIfTI files using an in-house developed script. After conversion, the following preprocessing steps were performed. First, functional scans were corrected for head motion with the realignment step. Then, coregistration of the functional scans was applied by coregistering the mean of the functional scans to the corresponding anatomy. Subsequently, normalization to a standard MNI brain template was performed (default SPM12). Last, functional scans were smoothed with an 8 mm full-width half maximum (FWHM) Gaussian kernel.

#### First-Level GLM Analysis

Design matrices were constructed for each subject. Onset times (per trial) and durations of the stimuli and instructions were derived from the E-prime logfiles. Each workload condition (0-back, 1-back, 2-back) was added as a condition. Instruction screens between the different workload conditions were added as a condition in the model. To correct for motion, the six motion parameters derived after the realignment step and their first derivatives were added as nuisance regressor. We applied a high pass filtering of 128 s. The following t-contrasts were computed: 2-back > 0-back, 2-back > 1-back, 1-back > 0-back, 2-back > baseline, 1-back > baseline, and 0-back > baseline.

#### Second-Level GLM Analysis

To assess group differences in brain activation, two-sample *t*-tests were performed for each first-level contrast. As the aim of the present study was explorative, we examined activation at the whole-brain level. To check for possible effects of depressive symptom severity, MDI scores were added as a group mean centered covariate in the second-level model. Both the main effect of the task (combining the two groups) and the task difference between groups were calculated. Additionally, the relation between the MDI covariate and brain activation (across groups), as well as the corresponding group difference were assessed. Group-level activation differences were considered significant at *p*-value < 0.05 family-wise error (FWE) corrected at cluster-level with an initial cluster-defining threshold of *p*-value < 0.001 uncorrected (default SPM12) (Eklund et al., 2016).

#### *Post hoc* Feature-Based ICA

The mass univariate approach (see previous section) reveals group-level differences. To investigate whether brain networks are associated with depressive symptom severity within the heartbreak group specifically, a *post hoc* feature-based Independent Component Analysis (ICA) was performed to identify brain regions that covaried across subjects (Calhoun and Allen, 2013). To this end, first-level 2-back > 0-back contrast images of the heartbreak group were used as inputs. The minimum description length (MDL) criterion was used to determine the optimal number of independent components (IC’s). Six IC’s were estimated using the infomax algorithm. Subject-specific spatial maps and beta courses were created using GICA back-reconstruction (Calhoun et al., 2001). IC’s were scaled to Z-scores. Estimated IC’s were visually inspected and obvious noise IC’s were omitted in further analyses. Spearman rank tests were performed to examine whether there was a relation between MDI scores and subject-specific loadings (i.e., values derived from the back-reconstruction step) on each IC. Correlations were considered significant at *p*-value < 0.05 (uncorrected).

## Results

### Study Population

The relationship group consisted of 23 men and 23 women with a relationship duration between 6 and 24 months (*Mdn* = 13.00, *IQR* = 9.00–19.00). 33 men and 37 women were included in the heartbreak group. Relationship duration prior to the breakup ranged between 6 and 81 months (*Mdn* = 20.00, *IQR* = 13.00–36.25) and time since breakup ranged between 0 and 5 months (*Mdn* = 2.00, *IQR* = 1.00–4.00). Information about age, educational level, current occupation and depressive symptom severity can be found in Table 2. The heartbreak group was slightly, but significantly, older than the relationship group (*U* = 1225.00, *Z* = −2.20, *p* = 0.028, *r* = −0.20). MDI scores were significantly higher in the heartbreak group (*U* = 1042.00, *Z* = −3.22, *p* = 0.001, *r* = −0.30).

**TABLE 2:** Characteristics of the relationship group and the heartbreak group.

		Relationship (*n* = 46)	Heartbreak (*n* = 70)
**Age [years, *Mdn (IQR)*]**		21.00 (20.00–23.00)	22.00 (21.00–24.00)
**Education (%)**	high school	82.6	61.4
	MBO	4.3	7.1
	HBO	6.5	10.0
	university	6.5	21.4
**Occupation (%)**	student	80.4	74.3
	student and working	17.4	18.6
	working	2.2	7.1
**MDI scores [*Mdn* (*IQR*)]**		7.00 (4.75–10.25)	9.00 (6.75–21.00)

### Behavioral Task Performance

The heartbreak group had a significantly higher accuracy (*U* = 1186.50, *Z* = −2.59, *p* = 0.010, *r* = −0.24) and a significantly lower RT (*U* = 1258.00, *Z* = −1.99, *p* = 0.047, *r* = −0.18) for the 1-back condition. With regard to the other workload conditions, no significant differences were found between the two groups. A more detailed overview of the behavioral task performance can be found in Supplementary Figure 1 and Supplementary Table 1.

#### Association Between Task Performance and Depressive Symptom Severity

In our total study sample, there was a negative correlation (*r_*s*_* = −0.22, *p* = 0.018) between MDI and RT for the 0-back condition. This effect seems to be driven by the heartbreak group, as a significant negative correlation between MDI and 0-back RT was found (*r_*s*_* = −0.28, *p* = 0.018) in this group, while in the relationship group no significant correlation between MDI and any task performance variable was found. Non-significant results are presented in Supplementary Table 2.

### fMRI Results

#### Main Effect of the Task

The total study sample displayed significant activation when contrasting the workload conditions to each other and to baseline. We especially observed activation in frontal and parietal regions. Table 3 shows the main effect of the task (activation when combining the heartbreak group and the relationship group).

**TABLE 3:** Peak activation when combining the heartbreak group and relationship group for each first-level contrast.

Contrast	Brain regions (cluster)	Cluster size	*p*_*FWE*_	*T*	MNI coordinates		
					x	y	z
**2-back > 0-back**	R middle frontal gyrus, L paracingulate gyrus	57159	<0.001	19.83	26	2	52
	R inferior temporal gyrus, R middle temporal gyrus	467	0.003	8.36	56	−54	−10
**2-back > 1-back**	L middle frontal gyrus, L R precuneus	43806	<0.001	15.86	−26	0	56
**1-back > 0-back**	R supramarginal gyrus, R superior frontal gyrus, R middle frontal gyrus	34935	<0.001	16.07	40	−40	42
	R inferior temporal gyrus, R middle temporal gyrus	1130	<0.001	10.15	56	−54	−8
	R intracalcarine cortex	355	0.013	5.03	24	−68	4
**2-back > baseline**	L paracingulate gyrus, L R supramarginal gyrus	49768	<0.001	19.69	−2	16	44
**1-back > baseline**	L supramarginal gyrus, L supplementary motor cortex, L superior parietal lobule	30012	<0.001	16.99	−46	−40	46
	R supramarginal gyrus, R superior parietal lobule, R inferior temporal gyrus	8457	<0.001	15.73	42	−38	42
**0-back > baseline**	L supplementary motor cortex, L supramarginal gyrus, L superior parietal lobule	13387	<0.001	14.22	−4	0	54
	L temporal occipital fusiform cortex, L middle temporal gyrus	1395	<0.001	10.53	−42	−60	−12
	R precentral gyrus, R frontal operculum	3076	<0.001	10.45	50	4	34
	R inferior temporal gyrus, R supramarginal gyrus	4845	<0.001	9.28	44	−58	−14
	R middle frontal gyrus	371	0.011	5.23	38	36	28

#### Group Differences in Brain Activation

**FIGURE 1:**
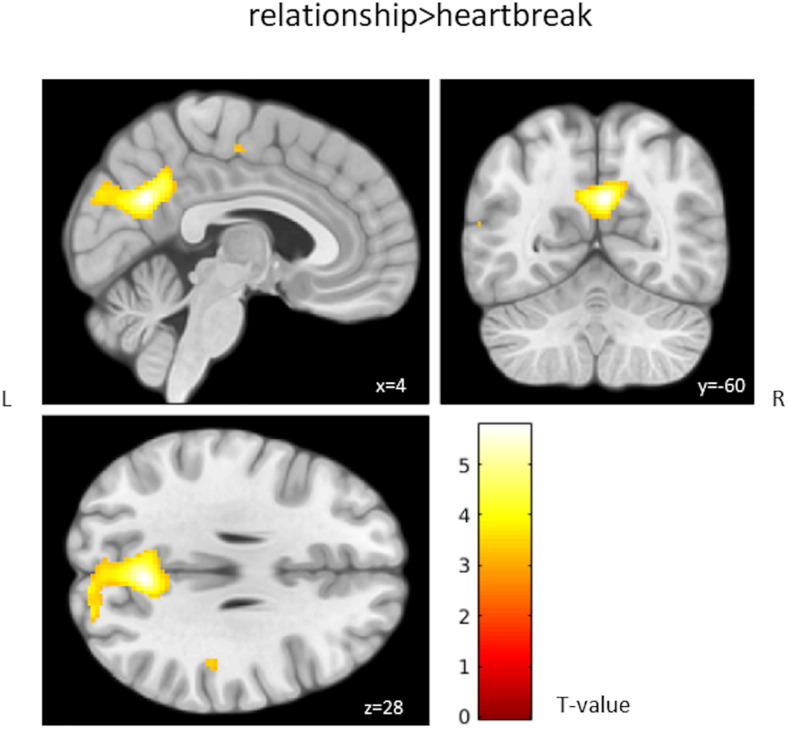
Group differences in activation during the 2-back > 0-back contrast. Activation above the T-threshold of 3.16 is displayed.

There was a significant interaction between group and 2-back > 0-back, 2-back > 1-back, and 2-back > baseline when contrasting relationship > heartbreak; the heartbreak group showed less activation in a cluster covering the precuneus. Table 4 shows peak activations when contrasting relationship > heartbreak for each first-level contrast and Figure 1 displays group differences in activation during the 2-back > 0-back contrast. No significant activation was observed when contrasting heartbreak > relationship for any of the first-level contrasts. Moreover, there was no interaction between group and covariate MDI.

**TABLE 4:** Peak activations when contrasting relationship > heartbreak for each first-level contrast.

Contrast	Brain regions (cluster)	Cluster size	*P*_*FWE*_	*T*	MNI coordinates		
					x	y	z
**2-back > 0-back**	L R precuneus	1060	<0.001	5.79	4	−60	28
**2-back > 1-back**	L R precuneus	602	0.002	4.55	0	−64	28
**1-back > 0-back**	no significant regions						
**2-back > baseline**	L R precuneus, R posterior cingulate gyrus	505	0.002	4.62	2	−62	28
**1-back > baseline**	no significant regions						
**0-back > baseline**	no significant regions						

#### Coactivated Brain Regions Within the Heartbreak Group

**FIGURE 2:**
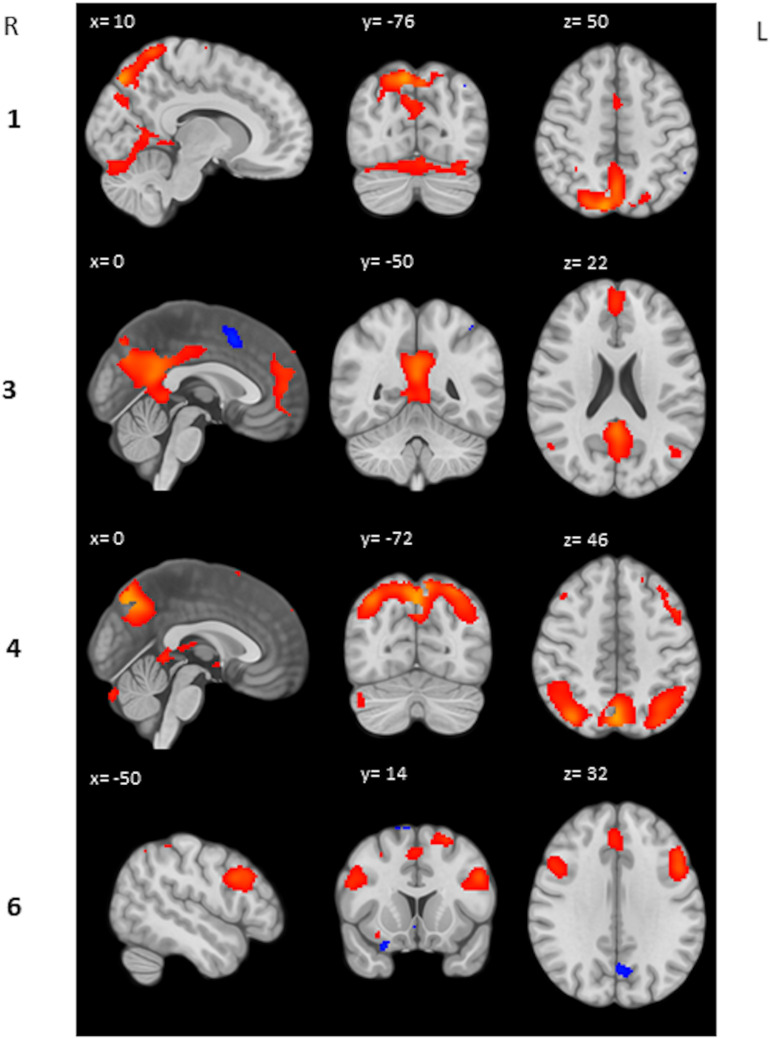
Peak activation IC’s. Respectively activation (red) and deactivation (blue) between *Z* = 2 and *Z* = 10 is displayed.

We performed a *post hoc* feature-based ICA on the 2-back > 0-back contrast images of the heartbreak group. Six IC’s were estimated and visually inspected. IC 2 and IC 5 were considered noise and therefore not included in further analyses. IC 1 represents among other brain regions the precuneus, anterior cingulate gyrus and supplementary motor cortex. IC 3 represents the posterior cingulate gyrus and paracingulate gyrus. IC 4 represents the precuneus and the lateral occipital cortex superior division. IC 6 represents among others the middle frontal gyrus and paracingulate gyrus. Table 5 shows peak coordinates and corresponding Z-scores for the remaining four IC’s. Figure 2 displays the peak activation for the four IC’s.

**TABLE 5:** Peak coordinates and corresponding Z-score for each IC.

IC	Brain region (peak)	*Z*	MNI coordinates		
			x	y	z
**1**	R precuneus	7.75	10	−76	50
**3**	L posterior cingulate gyrus	6.38	0	−50	22
**4**	L precuneus	8.02	0	−72	46
**6**	L middle frontal gyrus	5.24	−50	14	32

#### Association Between Depressive Symptom Severity and IC Loadings

**FIGURE 3:**
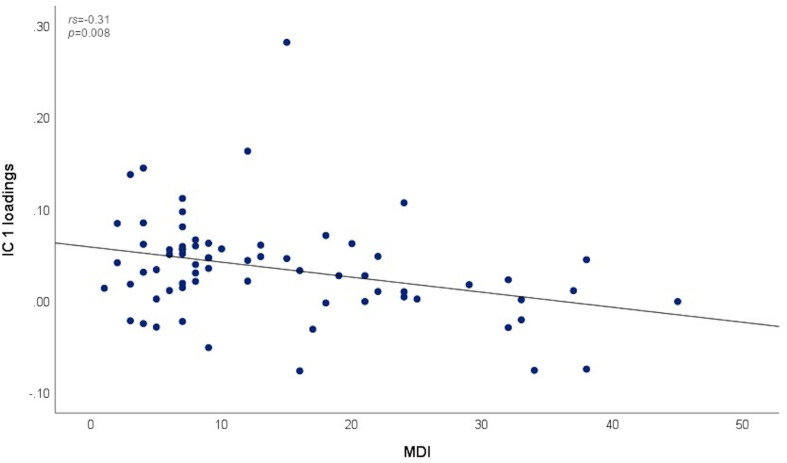
Association between MDI scores and subject-specific loadings on IC 1.

There was a negative correlation between the subject-specific loadings on IC 1 and MDI (*r_*s*_* = −0.31, *p* = 0.008, see Figure 3). No significant correlations were found between the other three IC’s and MDI (*r_*s*_* = −0.12, *p* = 0.312, *r_*s*_* = −0.14, *p* = 0.258, and *r_*s*_* = 0.07, *p* = 0.592 for, respectively, IC 3, IC 4, and IC 6).

## Discussion

In the present study, we investigated whether experiencing a romantic relationship breakup is associated with working memory alterations, both at the neural and behavioral level. Additionally, we investigated whether the possible association between working memory functioning and breakup is related to depressive symptom severity. This research question was motivated by an earlier study in our laboratory showing elevated depression scores, albeit subclinical, in a sample of individuals who experienced a romantic relationship breakup (Verhallen et al., 2019). Additional to elevated depression scores, stress-related cognitive alterations may occur in this population as well. The research aim of the present study was explorative, as previous studies have reported diverse results. Since both hyperactivation and hypoactivation at the neural level (Harvey et al., 2005; Rose et al., 2006; Matsuo et al., 2007; Fitzgerald et al., 2008; Sandström et al., 2012; Korgaonkar et al., 2013; Rodríguez-Cano et al., 2014), sometimes accompanied by inferior behavioral performance (Harvey et al., 2004; Ohman et al., 2007; Schoofs et al., 2008; Mackenzie et al., 2009), was found in stressed and/or depressed populations, we were not able to hypothesize on the direction of these breakup-related effects.

### Group Differences in Working Memory Functioning

We observed differences in brain activation between the heartbreak group and a comparison group consisting of individuals in a romantic relationship; the heartbreak group showed less activation in the precuneus when contrasting 2-back > 0-back, 2-back > 1-back, and 2-back > baseline. The precuneus is considered to be involved in various cognitive processes including memory retrieval and mental imagery (Fletcher et al., 1997). A meta-analysis by Owen et al. (2005) displayed the consistent involvement of parietal areas including the precuneus in performing the *n*-back task. The group-level activation result of our study is consistent with the study by Rodríguez-Cano et al. (2014) in which less activation of, among other brain regions, the precuneus was found in depressed patients when contrasting 2-back > baseline. In accordance with some studies in depressed (Harvey et al., 2005; Rose et al., 2006; Matsuo et al., 2007) and stressed (Sandström et al., 2012) populations, we did not find behavioral performance alterations during high workload (i.e., the 2-back condition) in the present study. Surprisingly, a better task performance (higher accuracy and lower reaction time) at moderate workload (i.e., the 1-back condition) was found in the heartbreak group compared to the relationship group in our study. A possible explanation for this result could relate to enhanced sensory information processing, as previously found in response to (acute) stress among healthy individuals (van Marle et al., 2009). So, the group-level analyses results of the present study suggest that breakup-related effects on working memory functioning are specifically present at the neural level during high workload, while behaviorally performing similar, and affect a brain region that previously was found to be important for working memory.

### Coactivated Brain Regions During High Workload Within the Heartbreak Group

We aimed to identify brain regions that covaried across subjects within the heartbreak group during high workload as our group-level activation analysis revealed differences between the two group with regard to precuneus activation, and it is known that the precuneus connects with several parts of the brain (Zhang and Li, 2012). In the present study, precuneus activation covaried with activation of the anterior cingulate gyrus and the supplementary motor cortex and independently with the lateral occipital cortex. The anterior cingulate gyrus is considered to be involved in, among others, processes related to attention and cognitive conflict and therefore seems to be important for task performance (Smith and Jonides, 1999). This network of coactivated regions, as found in the present study, is consistent with a study by Callicott et al. (1999) in which activation of a network of brain regions including parietal regions such as the precuneus, anterior cingulate, supplementary motor cortex, premotor cortex, and dorsolateral prefrontal cortex in response to increasing workload was found. Also, Sala-Llonch et al. (2012) revealed a working memory-related network using a data-driven approach, comprising the anterior cingulate gyrus, supplementary motor cortex, right middle frontal gyrus, frontal pole, left precentral gyrus, lateral occipital cortex, precuneus, and supramarginal gyrus. So, within the heartbreak group we identified coactivated brain regions during high workload consistent with previously found working memory-related networks. Subsequently, we aimed to investigate whether these identified brain networks are associated with depressive symptom severity.

### Relation Between Depressive Symptom Severity and Working Memory Functioning

We investigated whether depressive symptom severity is related to working memory functioning in our sample of subjects who recently experienced a stressful event (i.e., romantic relationship breakup), and reports on average elevated depression scores as well as a high intersubject variability (Verhallen et al., 2019). At the behavioral level, depressive symptom severity was found to be negatively associated with reaction time at low workload (i.e., the 0-back condition) in the present study, implying that subjects with more severe depression scores responded faster. At the neural level, we found specific group differences in brain activation that are consistent with a study in depressed patients (Rodríguez-Cano et al., 2014), although these were not driven by group differences with regard to depressive symptom severity, as there was no interaction between depression score and group in our study. However, subject-specific loadings on a network of brain regions, including the precuneus, anterior cingulate and supplementary motor cortex, were found to be negatively associated with depressive symptom severity in the heartbreak group, implying that this specific network is less represented in subjects with more severe depressive symptoms. Given that our heartbreak sample includes otherwise healthy individuals reporting elevated depression scores after a stressful event, this specific working memory-related network may be of importance with regard to the transition from healthy behavior, and corresponding brain activity, to depressive behavior during a disturbing period in life.

### Limitations

We have to take into account that, although depression scores were higher in the heartbreak group on average, only a quarter of the subjects reported symptoms corresponding to clinical depression (mild, moderate or severe) (Verhallen et al., 2019). Possibly, a higher sample size of subjects with severe symptoms would have made it possible to observe stronger effects of depressive symptoms on working memory functioning in our study. Though, it is noteworthy that even in our population of individuals experiencing relatively mild symptoms and without a psychiatric disorder, we find some altered neural activation during performance of a working memory task. Furthermore, as the design of our study was cross-sectional, possible baseline differences, unrelated to relationship status, between the heartbreak group and relationship group, could have been present and possibly could have influenced our outcomes. However, as the two groups are homogeneous in terms of age and education (young, highly educated), we expect, on average, minimal differences in baseline cognitive functioning. Regarding mood, we consider our finding of elevated depression scores in the heartbreak group likely to be related to the breakup, as both groups reported to be psychiatrically healthy. Though, prior differences in (predisposition to) mood problems cannot be ruled out completely. Moreover, one could argue that individuals in a romantic relationship are not a proper reference as stress hormone levels were found to be elevated during the first stage of a romantic relationship (Marazziti and Canale, 2004). However, in the present study we included people with a relationship duration of at least 6 months to control for this (Verhallen et al., 2019), and although we did not measure more direct indicators of stress such as cortisol levels, depression scores differed between our sample of individuals who experienced a breakup in the preceding 6 months and individuals currently in a romantic relationship, indicating stress-related mood disturbances. In addition, using individuals who have been single for a considerable period of time as a reference group would have been less optimal, as this group possibly is heterogeneous in terms of dating activity and associated stress levels (Chin et al., 2017). Last, a potential limitation of our study concerns the order of the fMRI session. It is known that inducing emotions inside the scanner can alter brain activity for a period of time (Servaas et al., 2013). During the first task of our fMRI session, prior to the working memory task, subjects were exposed to pictures of their (ex-) partner, as we intended to intensify the negative emotions and stress-related mood problems in the heartbreak group to examine break-up related effects on brain activity. As the subjects of the relationship group viewed pictures of their current romantic partner, this could have induced some (positive) emotions in this group as well, which could have affected their cognitive performance. This way, we were not able to measure working memory functioning in a pure way (i.e., regardless of any potential emotion inducements). However, we think that this has not harmed our main outcomes, as the two groups already, outside the experimental environment, substantially differ with regard to their emotional state.

### Conclusion

In the present study, we aimed to explore whether experiencing a romantic relationship breakup is associated with working memory alterations and whether this is related to depressive symptom severity. Taken together, our findings suggest that the effect of a romantic relationship breakup is, additional to elevated depression scores, associated with workload-dependent working memory alterations. Specifically, we found less precuneus activation and identified a working memory-related brain network within our heartbreak population that relates to depressive symptom severity. In an earlier study, based on questionnaire results, we proposed that the breakup of a romantic relationship can be used as an experimental model to examine stress-related depressive symptomatology in people without a psychiatric disorder (Verhallen et al., 2019). The findings of the present study strengthen this idea and show that this population can potentially be used to investigate the interplay between stress, cognitive functioning and depression. Especially, by conducting future longitudinal research, knowledge could be gained about the development of depression and related cognitive alterations during a disturbing period in life and potentially identify vulnerability factors for clinical depression.

## Data Availability Statement

The datasets presented in this article are not readily available because participants have not given consent to have their data publicly stored, and European Data Privacy Regulations (GDPR). However, de-identified behavioral and imaging data (NIfTI format) are available upon request. Requests to access the datasets should be directed to J-BM, j.b.c.marsman@umcg.nl.

## Ethics Statement

The studies involving human participants were reviewed and approved by the METc UMC Groningen. The patients/participants provided their written informed consent to participate in this study.

## Author Contributions

AV analyzed the data and wrote the original draft of the manuscript. RR and J-BM supported data analysis. J-BM and GH developed the experiment. RR, J-BM, and GH reviewed and edited the manuscript. GH obtained funding for the experiment. All authors contributed to the article and approved the submitted version.

## Conflict of Interest

The authors declare that the research was conducted in the absence of any commercial or financial relationships that could be construed as a potential conflict of interest.

## Footnotes

1 http://www.fil.ion.ucl.ac.uk/spm
