# Supplementary figures and images for: Working Memory Alterations After a Romantic Relationship Breakup
Source: Front Behav Neurosci. 2021 Apr 9;15:657264. doi: 10.3389/fnbeh.2021.657264 (PMC8062740; doi:10.3389/fnbeh.2021.657264)

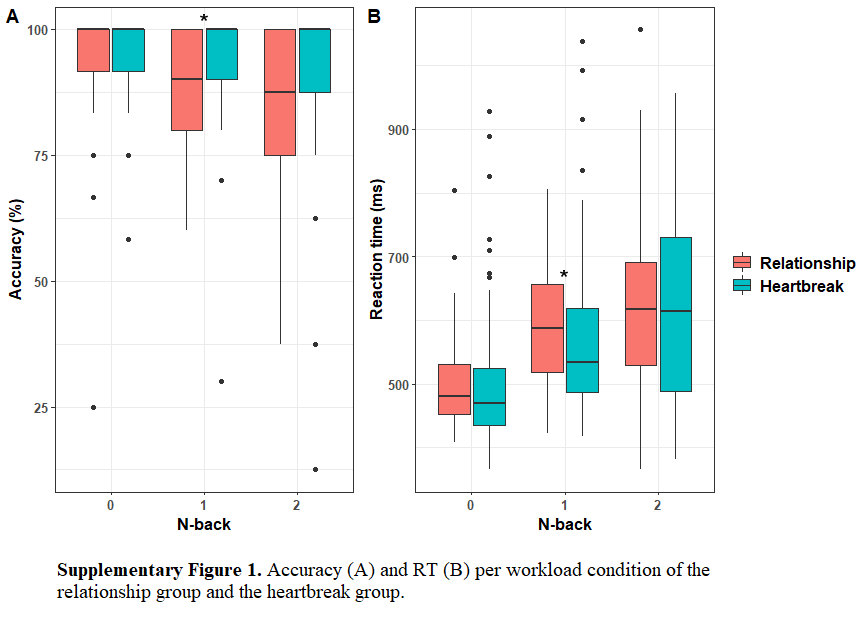

Supplement: Supplementary file 1 [file Image_1.TIF]
