# Supplementary material for: Working Memory Alterations After a Romantic Relationship Breakup
Source: Front Behav Neurosci. 2021 Apr 9;15:657264. doi: 10.3389/fnbeh.2021.657264 (PMC8062740; doi:10.3389/fnbeh.2021.657264)
Supplement: Supplementary file 2 [file Table_1.DOCX]

**Supplementary Table 1.** Accuracy and RT per workload condition of the relationship group and the heartbreak group.

|  | ***n*-back** | **Relationship (*n*=46)** | **Heartbreak (*n*=70)** | ***p*** |
| --- | --- | --- | --- | --- |
| **Accuracy (%, *Mdn* (IQR))** | **0** | 100.00 (91.67-100.00) | 100.00 (91.67-100.00) | 0.835 |
|  | **1** | 90.00 (80.00-100.00) | 100.00 (90.00-100.00) | 0.010* |
|  | **2** | 87.50 (75.00-100.00) | 100.00 (87.50-100.00) | 0.148 |
| **RT (ms, *Mdn (IQR))*** | **0** | 480.67 (452.33-533.17) | 469.38 (433.96-526.40) | 0.209 |
|  | **1** | 588.06 (516.32-660.00) | 534.55 (486.93-620.74) | 0.047* |
|  | **2** | 617.24 (529.18-697.30) | 613.88 (487.38-731.81) | 0.743 |
