# Supplementary material for: Working Memory Alterations After a Romantic Relationship Breakup
Source: Front Behav Neurosci. 2021 Apr 9;15:657264. doi: 10.3389/fnbeh.2021.657264 (PMC8062740; doi:10.3389/fnbeh.2021.657264)
Supplement: Supplementary file 3 [file Table_2.DOCX]

**Supplementary Table 2.** Correlations between MDI and the task performance variables.

| **Correlation** | **Relationship (*n*=46)** | | **Heartbreak (*n*=70)** | |
| --- | --- | --- | --- | --- |
|  | ***r_s_*** | ***p*** | ***r_s_*** | ***p*** |
| **0-back accuracy-MDI** | -0.08 | 0.614 | -0.17 | 0.172 |
| **0-back RT-MDI** | -0.05 | 0.736 | -0.28 | 0.018* |
| **1-back accuracy-MDI** | -0.15 | 0.313 | 0.02 | 0.881 |
| **1-back RT-MDI** | -0.04 | 0.801 | -0.19 | 0.118 |
| **2-back accuracy-MDI** | -0.18 | 0.231 | -0.03 | 0.824 |
| **2-back RT-MDI** | 0.06 | 0.694 | -0.19 | 0.121 |
